# EGFR mutation status in Tunisian non-small-cell lung cancer patients evaluated by mutation-specific immunohistochemistry

**DOI:** 10.1186/s12890-018-0706-5

**Published:** 2018-08-09

**Authors:** Zohra Mraihi, Jihen Ben Amar, Hend Bouacha, Soumaya Rammeh, Lamia Hila

**Affiliations:** 1 Genetic Department, Faculté de Médecine de Tunis, Université de Tunis El Manar, Tunis, Tunisia; 2 Pulmonary Department, EPS Charles Nicolle, Faculté de Médecine de Tunis, Université de Tunis El Manar, Tunis, Tunisia; 3 Pathological Anatomy and Cytology Department, EPS Charles Nicolle, Faculté de Médecine de Tunis, Université de Tunis El Manar, Tunis, Tunisia

**Keywords:** EGFR, Non-small-cell lung cancer, Mutation-specific immunohistochemistry, Targeted therapy

## Abstract

**Background:**

Screening mutations in epidermal growth factor receptor (EGFR) to analyze non-small-cell lung cancer (NSCLC) profile is the criterion to choose the best therapeutic strategy.

New Oncology guidelines recommend EGFR mutation analysis before prescribing tyrosine kinase inhibitors (TKIs) treatment.

Majority of lung cancer patients are diagnosed at advanced stages and generally only small biopsies materials are available for diagnostic and molecular characterization. The aim of this first work is to screen EGFR mutation status in Tunisian NSCLC by mutation-specific immunohistochemistry (IHC) and molecular biology, to estimate the relevance of proposing TKIs as a new therapeutic line.

**Methods:**

E746-A750 deletion and L858R mutations were screened in 50 unselected NSCLC formalin-fixed paraffin-embedded (FFPE) tissue samples. Mutation expression by IHC was evaluated by intensity and percentage of staining and correlated to patients’ data. DNA was extracted and EGFR mutations were analyzed by Sanger sequencing. Positive and negative controls were included for EGFR mutations in order to support the results.

**Results:**

Among our patients (48 men and 2 women) all adenocarcinoma (confirmed by histology and IHC with TTF1/Napsin A), 94% were smokers exceeding the tobacco risk threshold (at least 25 pack-years) and the women were none. 44% had EGFR mutation by IHC: 26% had simple mutation and 18% had concurrent mutation. All mutated cases were smokers except a woman who was none. Concurrent mutations patients exceeded 40 pack-years. 91.4% of IHC results were validated by molecular analysis (100% of negative and 85% of positive cases) showing either T > G (exon 21) or 2235–2249 del (exon 19).

**Conclusions:**

These preliminary results confirm the usefulness of IHC to detect EGFR mutations but the frequency of concurrent mutations doesn’t appear in favor of EGFR TKIs treatment. In fact, literature reports a significantly worse response compared to those with single mutation when treated by TKIs.

## Background

Lung cancer is a major cause of cancer-related mortality worldwide and is expected to remain a major health problem with increasing cases [1]. It is the leading reason of cancer death among men and the second in women, after breast cancer in the world [2]. In Tunisia, the lung is the main cancerous localization for male and an increasing incidence is observed with significant loss of life years in men (nearly a third of years of life lost) [3]. In 2015, World Health Organization (WHO) proposed new criteria for diagnosis and subclassification of lung cancer. These new guidelines were developed because two thirds of lung cancer diagnosis, presenting in advanced stages, are often established on small biopsy and cytology specimens [4–7].

This classification is not very different from historical one, since it also divides lung cancer in two groups: small and non-small-cell lung cancer (SCLC and NSCLC). The latter including: adenocarcinoma (ADC), squamous cell carcinoma (SCC), large cell carcinomas (LC), sarcomatoid carcinomas and mixed. Up to 85% of reported lung cancers are NSCLC. ADC accounts for more than 50% of these cases [8, 9].

One advance in cancer treatment is personalized medicine, where therapeutic is based on histology and genetic characteristics of each tumor. Last years, molecular mechanisms involved in lung cancer became better known and current treatments are now oriented toward efficient molecular-targeted therapies to improve pejorative prognosis [7, 10, 11].

Detection of driver mutations in NSCLC transformed thoracic oncology introducing oral small molecule tyrosine kinase inhibitors (TKIs) targeting specific EGFR mutations. EGFR mutations lead to strongest response to TKIs such as gefitinib [12, 13] and erlotinib [14]. Thus, evaluation of EGFR mutation status is important before undertaking therapy decision in advanced NSCLC. Hence, the importance, for pathologists to classify NSCLC into specific subtypes for determining eligibility to molecular testing and therapeutic strategies [9, 11, 15].

In Tunisia, in daily practice, we investigate only EGFR expression by classic IHC (total EGFR antibody). In the present study, we aimed to evaluate, for the first time in Tunisia, the use of EGFR mutation-specific antibodies for immunohistochemical (IHC) screening in NSCLC patients by comparing it with molecular analysis. IHC allows simultaneous analysis of level proteins expression and molecular characterization of tumor for specific molecular alterations and isn’t dependent on percentage of tumor cells in the sample unlike molecular tests, high costing and missing sensitivity as DNA is mainly obtained from FFPE tissues known to give poor quality DNA for sequencing [15, 16]. This study will evaluate IHC as a recourse analysis when molecular analysis is not possible and especially for small biopsies.

## Methods

### Study design

This retrospective study, which obtained ethical agreement, initially enrolled 50 unselected patients, 2 women and 48 men, from January 2010 to December 2014. Patients’ selection was based on the clinical diagnosis of NSCLC and clinical informations were obtained for each patient from the medical record database of Pneumology Department of EPS Charles Nicolle at Tunis. The study was done blindly without knowing histologic analysis results.

FFPE biopsies were collected from the tissue bank of Pathological Anatomy and Cytology Department. All cases were confirmed as NSCLC, by an experienced pathologist, based on hematoxylin and eosin (HE) staining according to the WHO criteria [7].

Lung biopsies collected were small but FFPE tissue sections analyzed by IHC presented at least 20% of tumor cells. The two most frequent mutations, E746-A750 del and L858R substitution respectively in exon 19 and 21 were screened.

### Immunohistochemical analysis

Histological classification and immunohistochemical staining were realized, for the 50 cases, upon 3 *µ*m FFPE sections HE stained after deparaffinization with xylene and rehydration through a graded series of ethanol concentrations.

For each patient, two slides were labeled, with antibody and protocol-specific bar codes, and loaded into a Benchmark GX (Ventana Medical Systems Inc) automated stainer. Slides were treated with Standard Cell Conditioning 1 (Ventana Medical Systems Inc) for 60 min. We used E746-A750 del (SP111) Rabbit Monoclonal Primary Antibody (ref 790–4650) and L858R (SP125) Rabbit Monoclonal Primary Antibody (ref 790–4649) from Ventana Medical Systems Inc.

Immunoreactivity was revealed with ultraView Universal DAB detection kit (Ventana Medical Systems Inc). The slides were counterstained with hematoxylin and bluing reagent for 4 min each. Positive and negative controls were run simultaneously. Negative control staining was performed by omitting the primary antibody and positive using lung adenocarcinoma known to express EGFR mutations.

Each slide was examined and scored, based on intensity and percentage of staining, independently by two pathologists based which were blinded to patients’clinicopathological and molecular data. In case of discordance, final result was done after approval of both.

### Scoring methodology

Immunoreactivity or IHC staining was scored according to the H-score (Histo-score) criteria, which assess the percentage (P) of positive cells (0–100%) multiplied by staining intensity (I) (0, no staining; 1, soft; 2, moderate; 3, strong; 4, very dark).

Final score varying from 0 to 400, [H = 1 x (% cells 1+) + 2 x (% cells 2+) + 3 x (% cells 3+) + 4 x (% cells 4+)] was calculated for each patient by two readers using the score with the maximum value [17].

### Molecular analysis

In order to confirm the molecular status of the analyzed cases by IHC, we perform molecular analysis, for both positive and negative cases, by Sanger sequencing to detect EGFR mutations. 15 cases were excluded from the molecular analysis, since there were no available residual FFPE tumor tissue samples. Indeed our work was retrospective and ethically we don’t have the right to exhaust the biopsies of patients.

Samples of DNA with known molecular status were analyzed in this study as controls: normal and EGFR mutation-positive presenting deletion in exon 19 and L858R point mutation in exon 21 (wild-type and mutated DNA cell lines: NCI-A549, NCI-H-1650 and NCI-H-1975, from Procell). DNA samples from 10 EGFR mutation-negative tumor lung tissue specimens, also negative by IHC, were enrolled in order to support our conclusions. DNA extraction was done using QIAamp DNA FFPE Tissue kit was used (Qiagen) according to the manufacturer’s protocol. The PCR was performed for the 2 exons 19 and 21 with specific primers [11, 17].

25 μL PCR reaction mixtures contain 100 ng DNA and 1.25 units Taq Polymerase. Amplification was done as follows: 33 cycles at 95 °C for 30s, 65 °C for 30s and 72 °C for 45 s followed by 7 min extension at 72 °C. EGFR gene was amplified by polymerase chain reaction using specific primers and DNA sequencing was performed using the ABI 3710 Genetic Analyzer (Applied Biosystems).

## Results

### Patients’ characteristics

Our cases included 48 men and 2 women, with a median age of 59.9 years (range 41–81 years). 3 never smokers and 47 former/current smokers. Histological analysis (TTF1 + Napsin A) revealed only ADC cases. Percentage of tumor cells was variable, but all had at least 20% and 74% of whom more than 30%.

Stage at the time of diagnosis was determined according to the tumor, node and metastasis (TNM) staging system: 37 patients were classified at stage IV, while 13 at IIIa or IIIb. Characteristics of patients are shown in Table 1. Metastases were present in 34.2% of the cases (bone, cerebral, hepatic ...). Most patients were treated with chemotherapy or surgery. No one benefited from targeted therapy. At the time of writing, 10 patients died (20%).

**Table 1:** Patients’ characteristics (*n* = 50)

Characteristics	n
Total	50
Age (year)	
Median	59,9
Range	41–81
Sex	
Male	48
Female	2
Smoking history	
Never-smoker	3
Former/current smoker	47
pTNM stage^a^	
IV	37
IIIa or IIIb	13
Type of treatment	
Surgery	4
Chemotherapy	21
Radiotherapy	3
Combined^b^	5
Transferred to private sector	17
Deceased cases	10

*pTNM* pathologic tumor-node-metastasis

^a^TNM classification 7th edition

^b^chemotherapy+radiotherapy

Survival estimation could only be achieved at 24 months, with a follow-up time from 1 to 24 months because of long time of medical care patients left to private sector. Overall survival was 6 months. Better survival was observed in patients aged less than 60 years.

### EGFR mutation-specific antibody IHC staining

**Fig. 1:**
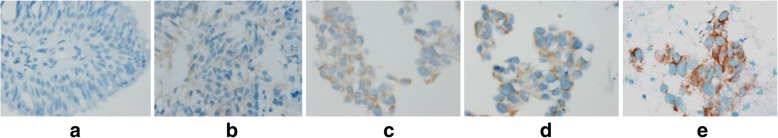
Immunostaining of tumor specimens with mutation-specific antibodies illustrating the scale of intensity of staining (original magnification, 40×); **a**: score 0; **b**: score 1; **c**: score 2; **d**: score 3 and **e**: score 4

**Fig. 2:**
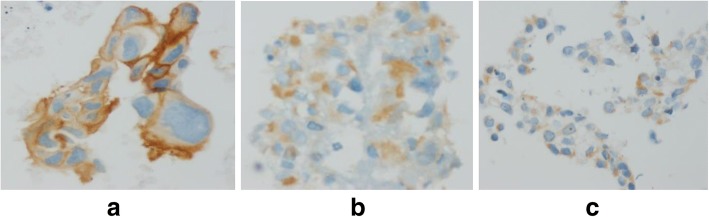
Membranous (**a**) / cytoplasmic (**b**) and mixte staining (**c**) (Original magnification, 40×)

Expression of E746-A750 del and L858R was evaluated in all 50 patients by IHC. The staining intensity was scored: blue: score 0, light brown: score 1, medium brown: score 2, dark brown: score 3 and very dark brown: score 4 (Fig. 1). Antibodies have distinct immunoreactivity for plasma membrane and cytoplasm of tumor cells. Cells showing membranous / cytoplasmic staining alone or in association were considered as positive and scored (Fig. 2).

### Immunoscoring

Amount of EGFR mutations was determined, for all patients, by calculating H-score, which evaluate heterogeneity of staining, based on estimation of staining area (%) per each intensity, since lung tumors are known to have heterogeneous mutational status.

Patients with only staining intensity 0 and 1+ were considered as negative for EGFR overexpression. The final H-score ranged from [0–240].

22/50 (44%) harbored an EGFR mutation by IHC and therefore 28 cases were negative.

26% (13/22) patients had simple mutation: 9 cases E746-A750 del and 4 cases L858R.

18% (9/22) patients had concurrent mutations E746-A750 del and L858R. 88.9% (8/9) of them were men. Only a woman who was non-smoker, stage IIIb had concurrent mutation.

67% (6/9) of patients, with concurrent exon 19 and 21 mutations, were at stage IV. 100% of men with concurrent mutation were smokers, 67% of whom were current and exceeding the risk threshold of lung cancer (at least 25 pack-years). Among former smokers, all exceeded 40 pack-years with variable consumption periods.

### Molecular analysis

**Fig. 3:**
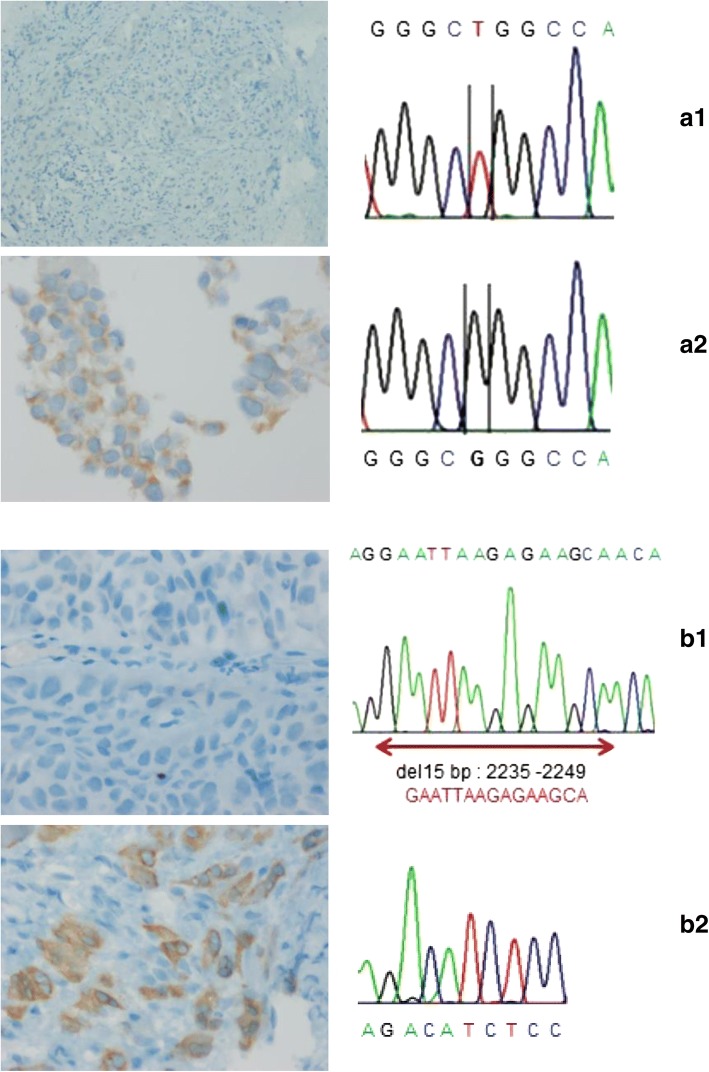
Concordance analysis IHC and DNA sequencing: *L858R*: **a1** (Patient 7): Left -- > negative IHC (Original magnification, 10×) / Right -- > normal electropherogram. **a2** (Patient 19): Left -- > positive IHC (Original magnification, 40×) Right -- > 2573 T > G point mutation in exon 21. *E746-A750*: **b1** (Patient 44): Left -- > negative IHC (Original magnification, 40×) Right -- > normal electropherogram. **b2** (Patient 33): Left -- > positive IHC (Original magnification, 40×) / Right -- > 2235–2249 del 15 bp

EGFR mutation detection was performed by PCR followed by Sanger sequencing for 35 patients (20 positive and 15 negative IHC cases) for which we could obtain DNA. Mutations were confirmed by sequencing for 17 of 20 positive cases by IHC (2 of the 22 positive IHC cases were not tested since we could not obtain DNA). 8 were concurrent and 9 simple mutations (7 had E746-A750 del and 2 had L858R mutation). One case of the concurrent mutations by IHC was only confirmed for a simple mutation (E746-A750 del). The most frequent EGFR mutation was E746-A750 del for exon 19 harboring 2235–2249 del 15 bp. For L858R mutated cases, 2573 T > G point mutation in exon 21 was detected (Fig. 3).

### Correlation IHC/molecular analysis

For the confirmed cases by molecular analysis (17 positive and 15 negative), the final H-score ranged from [50–240]: for simple mutation from [60–200] and for concurrent from [70–240]. IHC results were not confirmed by molecular analysis in 15% (3/20) of cases harboring an H-score less than 50. Then, they can be considered as false positive cases.

Majority of confirmed cases had H-score greater than 100 and 55% of concurrent mutations harbored for both a score superior to 110.

It is important to take in account the percentage of cells for each staining intensity (0 to 4+). All our IHC positive cases confirmed by sequencing presented a mix of staining intensity.

88.9% (8/9) of concurrent mutations and 72.7% (8/11) of simple mutations detected by IHC were confirmed, since 2 samples were not analyzed by sanger. Only one concurrent mutation was not confirmed and was classified as simple (E746-A750 del).

### Sensitivity and specificity of IHC-based method

In the nonmalignant tissues included in patient biopsies, no mutations of EGFR were observed both by IHC and sequencing. All negative EGFR IHC cases tested (15 cases) were confirmed by molecular analysis, after Sanger sequencing, and mutations were confirmed for 85% (17/20) of cases positive by IHC.

Sensitivity of IHC technique to detect EGFR mutation status compared to molecular analysis is concordant for 85% considering only positive cases by IHC and rise to 91.4% when we include negative IHC cases tested (32/35). Specificity to detect EGFR mutations by IHC was 100%: all IHC negative tissues from our patients tested (15 cases were we could obtain DNA) were confirmed by molecular analysis.

## Discussion

WHO Classification, 2015 of Lung Tumors, updated the diagnostic criteria in all lung cancer specialties: clinical, epidemiology, radiology, genetics, histology, cytology, IHC and molecular analysis [7].

Development of IHC provided better classification and reclassification of specific entities. Publications focused on the possibility of detecting mutated protein such as EGFR, BRAF… directly on lung cancer tissue by mutation-specific IHC with 92% of sensitivity which is comparable to DNA sequencing [15, 18].

But, there is a substantial need of data from several regions of the world, notably Africa, due to a lack of mutation testing. This is also the case in Tunisia.

We report the first Tunisian study carried out in 50 patients, enrolled from January 2010 to December 2014, by mutation-specific IHC to detect the most frequently EGFR mutations (exon 19 E746-A750 del and exon 21 L858R substitution), in FFPE tissues from small lung biopsies. Our patients were unselected, contrary to majority of works, analyzing EGFR mutations, done in patients with advanced stage or failing treatments (surgical or first-line chemotherapy).

Histological results (TTF-1/Napsin A) were consistent with ADC. 74% of them were at stage IV (Table 1). These results are concordant with literature.

A variable expression level of mutant EGFR proteins by immunoscoring was observed in the same tissue for all patients, indicating intratumoral heterogeneity. This observation is in line with literature which provides that abundance of EGFR mutation differs within each tissue [11]. For this purpose, we used H-score which takes in account this heterogeneity. There are two methods for immunoscoring, automated and manual, and two types of score, Q and H. We choose to use manual method to calculate H-score, although it is more difficult to compute, because Q-score ignores variable intensity of staining.

44% of our patients showed at least one EGFR mutation by IHC. This is concordant with the average mutation rate in many regions of Asia (Japan, Malaysia, Singapore,…). It is higher than overall Europe (15%), but similar to Germany and Turkey (up to 41%), noting that the number of studies by country remains relatively low [19–21].

Mutations average is variable for each study and each country. This variation can be explained by ethnicity which was not examined in most reported publications.

We choose to detect the most common NSCLC associated EGFR mutations, E746-A750 del and L858R, because they account together for 86 to 90% of total EGFR mutations (45% for E746-A750 del and 40–45% for L858R) [11, 17, 21–23].

Our result was based on H-score. IHC was considered positive, only for cases harboring high staining intensity (2+, 3+ and 4+). Patients with only 1+ were considered as negative for EGFR overexpression.

Among our mutated patients by IHC, we found 26% simple and 18% concurrent mutations. The most frequent mutation was in exon 19, 36% of our cases harboring E746-A750 del.

26% presented a substitution L858R in exon 21.

IHC results were confirmed in 91.4% of cases by molecular analysis. This is in accordance with literature [19].

IHC can be considered as an efficient specific tool to precise mutational status of patients.

Specificity to detect EGFR mutations was 100%. The relevance of sequencing has been validated by the use of negative and positive controls (cell lines).

Mutations types observed in Tunisian population are concordant with Indian and Moroccan studies which report E746-A750 del, as the most frequent mutation [19, 24].

18% of our patients harbored concurrent mutations by IHC. 88.9% of them were confirmed by sequencing. This result is little bit higher compared to literature (2.1 to 14%) [25–28]. This may be a Tunisian specificity, perhaps in relation with high frequency of smokers, noting that most studies enrolled Asian patients [20]. The only African study in literature is from Morocco and didn’t report concurrent mutations [24].

ADC histology and smoking history are the only significant independent predictors of EGFR mutation status [29].

Concurrent mutations were found in 43.7% of mutated men, all smokers exceeding 40 pack-years, and in one non-smoker woman. These profile and prevalence are not contradictory with literature. Although most studies reported a higher prevalence in non-smoker ADC women, in the PIONEER study more than 50% of patients with EGFR mutations were not non-smoker women [20, 25–30].

These results support EGFR mutation testing for all NSCLC patients.

In Tunisia, there are 1.7 million of smokers, aged from 10 to 70 years causing 10,000 cases of death each year (Plan for control of cancer in Tunisia 2015–2019). Tunisia is considered by WHO the most tobacco-consuming Arab country (35% of population).The latest statistical data highlights that most lung cancers are due to smoking in Tunisia and according to National Consumption Institute of Tunisia, most smokers consume smuggled cigarettes of poor quality, for economic reasons, multiplying by 11 the carcinogenic risk [31, 32].

Our results support this fact and pinpoint the pressing need for health authorities to inform and educate people relating to harmful effects of tobacco, focusing on primary prevention to discourage young people from taking up this practice but also supporting those wishing to stop smoking.

Actually in Tunisia, chemotherapy is the primary treatment for NSCLC. Our aim was to estimate the relevance of proposing TKIs for our patients as a new therapeutic line since patients harboring activating EGFR mutations can benefit from treatment with molecules like gefitinib and erlotinib [26, 30].

These preliminary results (majority of smokers and rate of concurrent mutations) don’t appear to support the use of TKIs for NSCLC in Tunisia, since smoking cigarettes (≥ 30 pack-years) is a negative predictive factor for TKIs treatment and the non-smoking mutated patients have the highest benefit from it [33, 34].

As well concurrent mutations lead to a worse response to TKIs treatment compared to single mutations (38% versus 89%, *p* < 0.001; ORR = 23.8%) [25, 26].

Only Zhang reported a better response in patients with co- mutations, treated with gefitinib or erlotinib, this contrary result may be explained by the small number of patients (3) treated [27].

Identifying subgroups of patients responding poorly to TKIs treatment may improve patients’ management [29]. Mechanisms of low response rate to TKIs are not clear, they may result from molecular conformation changes of EGFR tyrosine kinase domain caused by concurrent mutations [30].

To conclude, the good concordance between EGFR IHC and molecular sequencing data encourages the use of EGFR mutation-specific IHC as an easy and quick EGFR status “screening” approach. A negative IHC result is confident for the absence of EGFR mutation thus avoiding molecular analysis. By contrast, IHC positivity further requires gene sequencing to definitively assess the presence of EGFR mutation avoiding false positive cases.

In addition, although in the awareness of possible false positive, a relevant application of mutation-specific IHC is a better management of small size and/or low content tumor cells samples. It will avoid a second biopsy to obtain supplementary tissues to identify mutations especially for advanced cancer or tumor with limited cells [23]. Indeed, in daily practice, we are often confronted with little biopsies which will be included in paraffin for pathological analyzes. The latter will generally give small quantities with poor quality DNA, not always making molecular studies possible.

## Conclusion

There is a great need for further investigations to confirm the real contribution of EGFR mutation in lung cancer worldwide.

In Tunisia, this is the first report, which despite the small number of patients, gives a good concordance between molecular and IHC results, emphasizing the interest of setting up targeted IHC, in daily practice, to explore EGFR mutations in small biopsies of lung cancer but should be expanded to clarify relevance of TKIs treatment.

## Abbreviations

ADC: Adenocarcinoma
EGFR: Epidermal growth factor receptor
FFPE: Formalin-fixed paraffin-embedded
HE: Hematoxylin and eosin
H-score: Histo-score
IHC: Immunohistochemistry
LC: Large cell carcinomas
NSCLC: Non-small-cell lung cancer
SCC: Squamous cell carcinomas
SCLC: Small-cell lung cancer
TKIs: Tyrosine kinase inhibitors
TNM: Tumor, node and metastasis
WHO: World Health Organization
